# Improved protocol for plasma microRNA extraction and comparison of commercial kits

**DOI:** 10.11613/BM.2021.030705

**Published:** 2021-10-15

**Authors:** Harshini Sriram, Twinkle Khanka, Shweta Kedia, Priyanka Tyagi, Sitaram Ghogale, Nilesh Deshpande, Gaurav Chatterjee, Sweta Rajpal, Nikhil V Patkar, Papagudi G Subramanian, Sumeet Gujral, Syed Hasan, Prashant R Tembhare

**Affiliations:** 1 Hematopathology Laboratory, ACTREC, Tata Memorial Centre, Homi Bhabha National Institute, Kharghar, Navi Mumbai, India; 2 Hasan Laboratory, ACTREC, Tata Memorial Centre, Homi Bhabha National Institute, Kharghar, Navi Mumbai, India

**Keywords:** microRNA, plasma, commercial reagent kits

## Abstract

**Introduction:**

MicroRNAs are small, non-coding RNA molecules that are becoming popular biomarkers in several diseases. However, their low abundance in serum/plasma poses a challenge in exploiting their potential in clinics. Several commercial kits are available for rapid isolation of microRNA from plasma. However, reports guiding the selection of appropriate kits to study downstream assays are scarce. Hence, we compared four commercial kits to evaluate microRNA-extraction from plasma and provided a modified protocol that further improved the superior kit’s performance.

**Materials and methods:**

We compared four kits (miRNeasy Serum/Plasma, miRNeasy Mini Kit from Qiagen; RNA-isolation, and Absolutely-RNA MicroRNA Kit from Agilent technologies) for quality and quantity of microRNA isolated, extraction efficiency, and cost-effectiveness. Bioanalyzer-based Agilent Small RNA kit was used to evaluate quality and quantity of microRNA. Extraction efficiency was evaluated by detection of four endogenous control microRNA using real-time-PCR. Further, we modified the manufacturer’s protocol for miRNeasy Serum/Plasma kit to improve yield.

**Results:**

miRNeasy Serum/Plasma kit outperformed the other three kits in microRNA-quality (P < 0.005) and yielded maximum microRNA-quantity. Recovery of endogenous control microRNA *i.e.* hsa-miR-24-3p, hsa-miR-191-5p, hsa-miR-423-5p and hsa-miR-484 was higher as well. Modification with the inclusion of a double elution step enhanced yield of microRNA extracted with miRNeasy Serum/Plasma kit significantly (P < 0.001).

**Conclusion:**

We demonstrated that miRNeasy Serum/Plasma kit outperforms other kits and can be reliably used with a limited plasma quantity. We have provided a modified microRNA-extraction protocol with improved microRNA output for downstream analyses.

## Introduction

MicroRNA (miRNA) comprise a class of short (~22 nucleotides), endogenous non-coding RNA that are implicated in post-transcriptional regulation of protein expression (*1*). Several studies have highlighted the clinical utility of miRNA in the diagnosis and prognostication of disease (*2*-*4*). In particular, circulating or cell-free miRNA (cfmiRNA) have gained importance as non-invasive biomarkers for screening and monitoring of both solid and haematological malignancies. MicroRNA in biofluids like serum/plasma exhibit disease specificity and has remarkable stability (*5*-*7*). In cell-free conditions, miRNA is protected from endogenous RNase activity by its association with vesicles or proteins such as Ago2 or other RNA-binding proteins (*8*-*10*). Therefore, cfmiRNA offer an unbiased and viable alternative to existing strategies to ascertain disease condition.

However, the reliable application of cfmiRNA in clinics is still limited due to the lack of robust reproducibility in clinical samples. One of the major challenges of the clinical applications of cfmiRNA studies is the inadequate understanding of many preanalytical variables, including sample storage and RNA isolation from small volumes of clinical samples. To date, several kits are commercially available to facilitate the rapid extraction of cfmiRNA. However, studies evaluating the effectiveness of commercially available kits with clinical specimens are scarce (*11*-*13*). Hence, the present study was aimed to assess the performance of four commonly available commercial kits for isolation of cfmiRNA from small volumes of human plasma samples. We also modified the manufacturer’s protocol for the kit with superior performance to improve the miRNA yield further.

## Materials and methods

### Subjects

**Figure 1:**
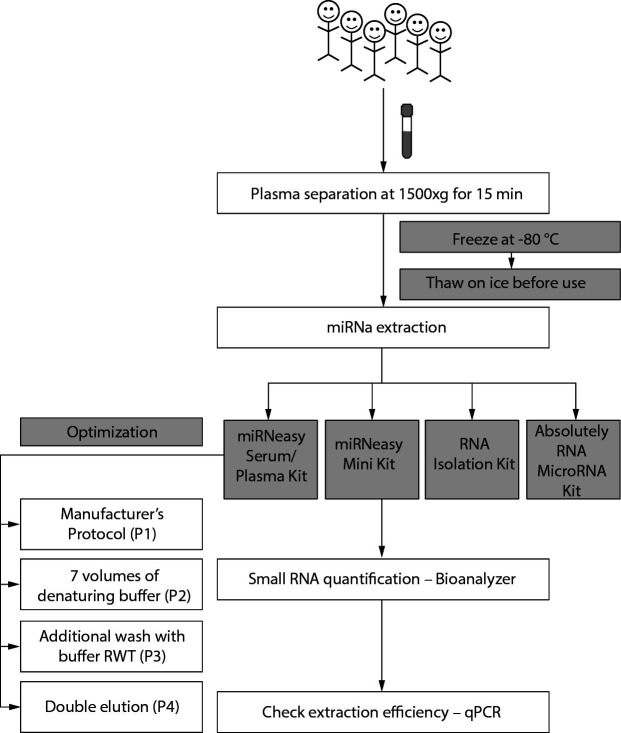
Schematic view of the experimental design and workflow. miRNA - microRNA. qPCR - real-time polymerase chain reaction.

This was a prospective study focusing on the standardization of miRNA extraction from plasma samples collected with EDTA anticoagulant. Whole blood samples were collected from 30 healthy donors. Written informed consents were obtained from all volunteers. The experimental design implemented in this study has been illustrated in Figure 1.

According to the protocol approved by the Institutional Ethics Committee (Tata Memorial Centre – Institutional Ethics Committee III), approximately 3 mL of whole blood samples were collected in 5 mL K2EDTA vacutainer tubes (BD vacutainer, Becton Dickinson, Plymouth, UK). The samples were spun at 1500xg for 15 min at 4 ºC to separate plasma. Without disturbing the buffy coat, the clear supernatant was transferred to a fresh 2 mL cryotube (Tarsons, Saltlake, Kolkata, India) and inspected for haemolysis. The samples with no visual signs of haemolysis were immediately frozen at - 80 ºC for 4-6 months.

### Materials

We assessed four commercially available micro/small RNA extraction kits for their quality, yield, extraction efficiency, and cost-effectiveness. The study included miRNeasy Serum/Plasma kit (Qiagen, Hilden, Germany), miRNeasy Mini kit (Qiagen, Hilden, Germany), RNA isolation kit (Agilent Technologies, Santa Clara, USA), and Absolutely RNA MicroRNA kit (Agilent Technologies, Santa Clara, USA). RNeasy MinElute Cleanup Kit (Qiagen, Hilden, Germany) was obtained for use with miRNeasy Mini kit. For use as spike-in or exogenous control 5’-phosphorylated synthetic RNA oligo *C. elegans* miRNA cel-miR-39-3p (Sigma-Aldrich, St. Louis, Missouri, USA) was obtained. Agilent Small RNA kit (Agilent Technologies, Santa Clara, USA) was procured for miRNA quantification. The efficiency of each protocol was evaluated with TaqMan technology-based real-time polymerase chain reaction (qPCR) using TaqMan Advanced miRNA cDNA Synthesis kit, TaqMan Fast Advanced Master Mix, and TaqMan Advanced miRNA Assays (Applied Biosytems, Thermo Fisher Scientific, Waltham, USA).

### Methods

#### Plasma preparation

Plasma samples were allowed to thaw completely on ice and centrifuged at 3000xg for 5 min at 4 °C to remove any cryoprecipitate. A total of 200 μL of plasma was utilized *per* extraction.

#### miRNA extraction

RNA extraction was performed in accordance with the manufacturer’s protocol for each of the four kits. For all miRNA isolations, 5 pM (5.44 x10^8^ copies or 0.54 x10^8^ copies/µL) of the synthetic cel-miR-39-3p was spiked into the sample before extraction.

##### Kit 1. miRNeasy Serum/Plasma kit

Briefly, 5 volumes of QIAzol lysis Reagent (Qiagen, Hilden, Germany) were mixed with 200 μL of plasma and incubated at room temperature for 5 min. Post incubation, 5 pM cel-miR-39-3p was spiked into the homogenate. Subsequently, 200 μL of chloroform was added to the lysate and incubated for 2 min at room temperature. The solution was centrifuged for 15 min at 12,000xg at 4 °C. The upper aqueous layer was transferred to a fresh microfuge tube and mixed with 1.5 volumes of 100% ethanol. The solution was transferred into an RNeasy MinElute spin column (Qiagen, Hilden, Germany) and centrifuged at 8000xg for 15 seconds at room temperature. The spin column was washed twice, once each with the two wash buffers provided (RWT and RPE), and then with 80% ethanol. Finally, miRNA was eluted in 14 μL RNase-free water.

##### Kit 2. miRNeasy Mini kit

Briefly, 700 μL QIAzol lysis Reagent was mixed with 200 μL of plasma and incubated for 5 min at room temperature. A total of 5 pM of cel-miR-39-3p was spiked into the homogenate. Afterwards, 140 μL of chloroform was added to the lysate and incubated for 2 min at room temperature. The solution was centrifuged for 15 min at 12,000xg at 4 °C. The upper aqueous phase was transferred to a fresh microfuge tube and mixed with equal volume of 70% ethanol. The solution was transferred into an RNeasy Mini column and centrifuged at 8000xg for 15 sec. The flow-through was collected into a fresh microfuge tube and mixed well with 0.65 volumes of 100% ethanol. The sample was transferred into a fresh RNeasy MinElute spin column and centrifuged at 8000xg for 15 sec. The spin-column was subjected to two washes, once with each of the buffers provided (RWT and RPE) and then with 80% ethanol. Subsequently, miRNA was eluted in 14 μL RNase-free water by centrifugation of the spin column at 8000xg.

##### Kit 3. RNA isolation kit

The RNA isolation kit follows a column-free extraction protocol based on alcohol precipitation of RNA. In short, 200 μL of plasma was incubated with 2 mL of denaturing solution (solution D). A total of 5pM of cel-miR-39-3p was spiked into the homogenate. This was followed by the addition of 200 μL of 2M sodium acetate (pH 4.0), 2 mL of phenol (pH 5.3-5.7), and 400 μL of chloroform-isoamyl alcohol to the lysate. The solution was mixed well and centrifuged for 15 min at 12,000xg at 5-10 °C. The upper aqueous phase was transferred to a fresh centrifuge tube. One volume of isopropanol was added to the aqueous phase, mixed well, and centrifuged for 30 min at 10,000xg. The pellet was washed with 75% ethanol, air-dried, and resuspended in 50 μL RNase-free water.

##### Kit 4. Absolutely RNA MicroRNA kit

Briefly, 200 μL of lysis buffer containing β-mercaptoethanol was added to 200 μL of plasma. The contents were mixed well to homogenize. A total of 5pM of cel-miR-39-3p was spiked into the homogenate, followed by the addition of one volume of phenol-chloroform (1:1). The solution was mixed well and centrifuged for 4 min at maximum speed at room temperature. The upper aqueous phase was transferred to a fresh microfuge tube. One volume of chloroform-isoamyl alcohol (24:1) mixture was added and centrifuged for 3 min at maximum speed. The resulting aqueous phase was transferred into a prefilter spin cup provided and centrifuged for 3 min at maximum speed. The filtrate was collected, mixed with 1.25 volumes of 100% ethanol, and transferred into an RNA-binding spin cup. The RNA-binding cup was centrifuged for 1 min at full speed. The spin cup was washed with Low-Salt buffer provided and subjected to on-column DNA digestion for 15 min at 37 °C. The spin cup was then washed with the High-Salt buffer provided. Furthermore, 50 μL of RNase-free water was added onto the column and incubated on the bench-top for 1 min. Finally, miRNA was eluted by centrifugation of the spin column at maximum speed for 1 min.

#### Modifications to the miRNeasy Serum/Plasma kit

Modifications to the manufacturer’s protocol (Protocol1/P1) were made to optimize miRNA extraction. Cell free miRNA was extracted from the plasma of 20 healthy donors collected and frozen previously. All extractions were performed in duplicates to access reproducibility and intra-sample variability. The protocol was modified at different stages. First, the ratio of denaturing buffer to plasma volume was increased from the suggested 5:1 to 7:1 (Protocol2/P2), with 7 volumes of denaturing reagent being the maximum holding capacity of a 2 mL microfuge tube per 200 μL of plasma used. Next, the column was subjected to an increased number (2x) of washes with the wash buffer (buffer RWT) prior to the elution step (Protocol3/P3). Finally, the elution step of the protocol was modified by incorporating a double elution protocol (Protocol4/P4). A 10 min bench-top incubation with RNase-free water was allowed before miRNA elution. The eluent was added onto the column again and eluted a second time to ensure efficient elution of all membrane-bound RNA.

#### Small RNA quantification

The concentration of miRNA in the elute was measured on a Bioanalyzer 2100 (Agilent Technologies, Santa Clara, USA) using the Agilent Small RNA kit. Instrument set-up, reagent preparation, and small RNA assay were performed according to the manufacturer’s instructions. The small RNA region and miRNA region were arbitrarily defined as fragments between 10-150 nucleotides and 14-30 nucleotides, respectively.

Given the difference in elution volumes across the kits being evaluated, data is represented as the total amount of miRNA. Total amount of miRNA per extraction was calculated as follows: Total amount of miRNA = concentration x volume in mL cDNA synthesis.

Reverse transcription (RT) was performed using TaqMan Advanced miRNA cDNA Synthesis Kit according to the manufacturer’s protocol. Briefly, 5 ng of the extracted RNA was reverse transcribed using universal RT primers supplied in the kit. The cDNA was stored at - 20 ºC until further use.

#### Real-time PCR

MicroRNA quantitation was carried out using the TaqMan Advanced miRNA Assays and TaqMan Fast Advanced Master Mix on a QuantStudio real-time PCR system (Applied Biosystems, Thermo Fisher Scientific, Waltham, USA). Real time PCR reactions were set up for the spike-in control and four endogenous miRNA controls - hsa-miR-24-3p, hsa-miR-191-5p, hsa-miR-423-5p and hsa-miR-484. Individual qPCR assays were performed in triplicates with a total reaction volume of 10 μL. The assays were performed according to the manufacturer’s instructions.

### Statistical analysis

Statistical analyses were performed using GraphPad Prism version 8.0 for Windows (GraphPad Software, San Diego, USA). The results were expressed as the median and interquartile range (IQR). The difference between quality (percentage purity) and quantity of the miRNA yield and endogenous control recovery between the different kits were evaluated using one way ANOVA. Tukey’s multiple comparison test was used *post hoc* for multiple testing and P values were reported.

A paired sample t-test was performed to compare each protocol modification to that of the manufacturer’s protocol. Intra-assay variability was determined with the variation (CV) coefficient for the manufacturer’s protocol and the three modifications and expressed as a percentage (CV%). The level of significance for all tests performed was defined as P < 0.05.

## Results

### miRNA quality and quantity assessment

Our data revealed that the quality of miRNA from the miRNeasy Serum/Plasma kit was better than the other 3 kits (P < 0.005). The quality of miRNA extracted using the RNA Isolation kit and Absolutely RNA MicroRNA kit from Agilent Technologies showed a significant decrease (P < 0.001) in comparison with the miRNeasy Serum/Plasma kit. The miRNeasy Mini kit and Absolutely RNA MicroRNA kit produced yields of comparable quality, with no significant difference from one another (P = 0.661). The results of the comparison of miRNA quality isolated from the kits are given in Table 1.

**Table 1:** Qualitative and quantitative comparison of miRNA extraction kits

**Variable**	**miRNeasy Serum /Plasma kit**	**miRNeasy Mini kit**	**RNA Isolation kit**	**Absolutely RNAMicroRNA kit**	**P-value**
miRNA quality (% miRNA in small RNA)	77.50 (74.00-83.00)	72.00 (68.50-76.25)	42.50 (34.75-50.25)	73.50 (68.00-77.25)	< 0.001*
					< 0.001^†^
					< 0.001^‡^
					< 0.001^§^
					0.661^ǁ^
					< 0.001^¶^
miRNA yield (total amount of miRNA in ng)	19.20 (16.80-20.30)	17.30 (14.10-18.10)	16.10 (14.00-17.70)	18.20 (17.40-19.20)	< 0.001*
					< 0.001^†^
					< 0.198^‡^
					< 0.237^§^
					< 0.001^ǁ^
					< 0.001^¶^
Results are represented as median and interquartile range (Q1-Q3). The differences between the four kits were estimated using one way ANOVA. Multiple comparisons were made using Tukey’s test. For all tests, P < 0.05 was considered significant. *Comparing miRNeasy Serum/Plasma and miRNeasy Mini kit; ^†^comparing miRNeasy Serum/Plasma and RNA Isolation kit; ^‡^comparing miRNeasy Serum/Plasma and Absolutely RNA MicroRNA kit; ^§^comparing miRNeasy Mini and RNA isolation kit; ^ǁ^comparing miRNeasy Mini and Absolutely RNA MicroRNA kit; ^¶^comparing Absolutely RNA MicroRNA kit and RNA Isolation.					

Similarly, we observed that the miRNeasy Serum/Plasma kit yielded maximum miRNA upon elution. There was no significant difference in miRNA yields between this and the Absolutely RNA MicroRNA kit (P = 0.198). We did not observe a significant difference in miRNA quantity between Qiagen’s miRNeasy Mini kit and RNA Isolation kit from Agilent Technologies (P = 0.237). However, the miRNeasy Serum/Plasma kit yielded higher quantities of miRNA (P < 0.001) in comparison to the abovementioned kits (Table 1). Additionally, the two extraction kits from Agilent Technologies also exhibited a significant difference in the total amount of miRNA extracted (P < 0.001).

### Detection of endogenous control miRNA

All four kits recovered the spike-in miRNA and endogenous control miRNAs. However, the extent of recovery varied considerably. Detection of endogenous miRNA is represented by their normalized quantitation cycle (Ct) value across the 4 kits (Table 2). Our results indicated that the overall recovery of the control miRNAs was greater with the miRNeasy Serum/Plasma kit. Additionally, the RNA Isolation kit demonstrated the lowest recovery as reflected by higher Ct values for the endogenous miRNAs. The results of the comparison of endogenous miRNA recovery between the kits are presented in Table 2.

**Table 2:** Endogenous miRNA recovery comparison of miRNA extraction kits

**Variable**	**miRNeasy Serum/ Plasma kit**	**miRNeasy Mini kit**	**RNA Isolation kit**	**Absolutely RNA MicroRNA kit**	**P-value**
Recovery of hsa-miR-24-3p (Ct)	18.26 (17.27-18.84)	20.46 (19.41-21.85)	21.46 (20.19-22.84)	19.11 (18.30-19.85)	< 0.001* < 0.001^†^ 0.013^‡^ 0.012^§^ 0.001^ǁ^ < 0.001^¶^
Recovery of hsa-miR-191-5p (Ct)	19.33 (18.18-20.63)	20.27 (19.13-21.61)	21.58 (20.43-22.82)	20.18 (18.46-20.63)	0.008* < 0.001^†^ 0.483^‡^ 0.010^§^ 0.269^ǁ^ < 0.001^¶^
Recovery of hsa-miR-423-5p (Ct)	18.72 (17.99-20.83)	19.81 (18.79-21.50)	20.47 19.07-21.54	19.97 18.27-20.90	0.142* 0.004^†^ 0.521^‡^ 0.528^§^ 0.861^ǁ^ 0.146^¶^
Recovery of hsa-miR-484 (Ct)	18.35 17.28-19.21	20.15 19.26-21.98	21.10 19.33-22.60	19.12 18.00-20.04	< 0.001* < 0.001^†^ 0.110^‡^ 0.856^§^ 0.001^ǁ^ < 0.001^¶^
Results are represented as median and interquartile range (Q1-Q3). The differences between the four kits were estimated using one way ANOVA. Multiple comparisons were made using Tukey’s test. For all tests, P < 0.05 was considered significant. *Comparing miRNeasy Serum/Plasma and miRNeasy Mini kit. ^†^comparing miRNeasy Serum/Plasma and RNA Isolation kit. ^‡^comparing miRNeasy Serum/Plasma and Absolutely RNA MicroRNA kit. ^§^comparing miRNeasy Mini and RNA isolation kit. ^ǁ^comparing miRNeasy Mini and Absolutely RNA MicroRNA kit. ^¶^comparing Absolutely RNA MicroRNA kit and RNA Isolation. Ct – quantification cycle value normalized using cel-miR-39-3p spike-in control.					

### Optimization of miRNeasy Serum/Plasma kit

Initial results suggested that miRNeasy Serum/Plasma kit (Qiagen, Hilden, Germany) outperformed the other isolation kits and provided superior quality elute with a higher quantity of miRNA. The recovery of endogenous control miRNA was also better with the miRNeasy Serum/Plasma kit than the other three tested. We hypothesized that a few modifications to the manufacturer’s protocol for miRNeasy Serum/Plasma kit could further improve miRNA yield. Hence, we implemented a few modifications as described in the “Materials and methods” section.

The results of a comparison between the modified protocols (P2-P4) with the manufacturer’s original protocol (P1) are given in Table 3. Intra-sample variability was calculated for quality and quantity of miRNA extracted by the original and modified protocols and is expressed as average CV%. The quality of miRNA isolated from the original protocol (P1) showed variability (CV%) of 6.92%, and that of modified protocol P2 was 5.67%, P3 - 7.06%, and P4 - 5.04%. There was no significant difference between the quality of miRNA extracted from P1 and P2 (P = 0.061). In comparison, P3 had a marked decrease in quality (P < 0.001) while P4 improved the quality of the yield significantly (P = 0.007).

**Table 3:** Assessment of miRNeasy Serum/Plasma protocol amendments

**Protocol**	**P1**	**P2**	**P3**	**P4**	**P-value**
miRNA quality (% miRNA in small RNA)	79.00 (73.13-81.75)	77.25 (72.00-81.38)	71.00 (68.25-74.75)	81.25 (76.13-83.00)	0.061*
					< 0.001^†^
					0.007^‡^
miRNA yield (Total amount of miRNA in ng)	19.25 (16.93-20.75)	18.25 (17.05-19.68)	17.80 (16.88-19.25)	21.35 (18.40-22.58)	0.189*
					0.003^†^
					< 0.001^‡^
Protocol P1 is the manufacturer’s original protocol for miRNeasy Serum/Plasma kit. P2–P4 are modified protocols tested to optimize miRNA extraction from the kit. Results are represented as median and interquartile range (Q1-Q3). The differences between the four kits were estimated using paired sample t-test. For all tests, P < 0.05 was considered significant. *Comparing P1 and P2. ^†^comparing P1 and P3. ^‡^comparing P1 and P4.					

A similar trend was noticed in the relative variability between the quantity of miRNA extracted using original and modified protocols. However, we observed a greater variability across all four protocols. Average CV% for the quantity of miRNA isolated from P1 was 7.62%, P2 - 8.81%, P3 - 10.54%, and P4 - 6.29%. In comparison we noticed no significant difference in the quantity of miRNA yielded from P1 and P2 (P = 0.189). On the other hand, P3 yielded lower quantities of miRNA (P = 0.003) while P4 significantly improved the miRNA yield upon extraction (P < 0.001). Thus, P4-modification demonstrated the lowest variability in the quality and quantity of miRNA extracted.

## Discussion

In this study, we assessed four commercially available RNA isolation kits for their efficiency in extracting good quality and quantity of miRNA.

The sensitivity of downstream assays of miRNA, including qPCR and microarray, is largely affected by residual salts from denaturing and wash buffers used in the extraction process. Due to the low abundance of miRNA in plasma, selecting an ideal miRNA isolation kit becomes crucial. In this study, the RNA Isolation kit from Agilent Technologies demonstrated relatively lower performance across all parameters evaluated. This could be partly attributed to the duration of extraction or even residual salt contamination. However pure the reagents may be, prolonged RNA isolation protocols elevate the risk of RNase contamination, leading to degradation of miRNA. Additionally, this kit does not employ a filtering agent, such as silica gel or beads, to ensure adequate removal of residual chaotropic salts from buffers used. It relies on the principles of organic extraction and thus, may not be a very compelling choice for small RNA studies. On the other hand, our findings suggested that the miRNeasy Serum/Plasma kit provides relatively higher quality and quantity of miRNA when compared to other kits. The ease of use and short duration for extraction provide further advantages.

The quality of miRNA is a crucial factor as it impacts downstream analyses, especially quantification. The RNA profile of plasma is depleted of traditional markers of RNA quality and integrity, including large RNA species such as messenger RNA and the ribosomal RNA subunits. The RNA pool from peripheral blood is dominated by miRNA, fragmented mRNA, small nucleolar RNA, and other small RNA species (*14*). Hence, the 28s to 18s rRNA ratio is not reliable in the assessment of cfmiRNA quality. In our study, this limitation was overcome by using the proportion of miRNA in total small RNA as an indicator of cfmiRNA quality. For this, the size range of small RNA and miRNA were arbitrarily defined as fragments between 10-150 and 14-30 nucleotides in length respectively. A higher proportion of miRNA in small RNA indicated a greater quality of RNA isolated.

One of the challenges of plasma miRNA studies is that despite being one of the dominant RNA species in liquid biopsies, miRNA yields from plasma are much lower than solid tissues. This limitation demands kits that can maximize miRNA yield from minimal input volumes of serum or plasma.

Most recommended protocols provided with the commercial kits are generic and require additional optimization to be reliably used. Optimization of protocols is required to obtain high-quality, high-yielding miRNA from small volumes of input samples in reproducible manner. The miRNeasy Serum/Plasma kit showed superior performance across parameters tested compared to the other kits evaluated in this study. Hence, we hypothesized that additional optimization of the protocol provided with miRNeasy Serum/Plasma kit could further improve the resulting miRNA yield. We assessed an improvisation to the quick-start protocol from the kit for plasma miRNA extraction. We observed that incorporating a double-elution step with an on-column incubation of 10 minutes leads to a significant increase in the concentration of miRNA extracted. This increase in concentration also reflected positively on the quality of miRNA extracted.

Previous studies suggested that DNA contamination in extracted miRNA could interfere with miRNA detection (*15*). Hence, we further evaluated the effect of possible DNA carryover on the detection of endogenous control miRNA extracted using modified (P4) protocol for miRNeasy Serum/Plasma kit by TaqMan technology-based qPCR reactions (data not shown). DNA carryover was quantified using Qubit Double-Stranded DNA High Sensitivity Assay kit (Thermo Fisher Scientific, Waltham, USA). We noticed DNA carryover in the range of 0.06-2.15 ng/µL. To evaluate DNA interference in the quantification of miRNA we compared the detection of endogenous control miRNAs with and without reverse transcription. We observed that signal was produced against all 4 miRNAs only upon reverse transcription. Endogenous miRNAs were not detected in the RT control (subject to all steps of cDNA synthesis but for the addition of Reverse Transcriptase), suggesting that a DNase I digestion is not essential under these circumstances.

Overall, the results of our study were in accordance with findings reported by Inés Moret *et al*., Kathryn Wright *et al*., and Ari Meerson *et al.* (*16*-*18*). These studies also compared various miRNA isolation kits and demonstrated that the miRNeasy Serum/Plasma kit from Qiagen produces the most favorable results. However, with the exception of the miRNeasy Serum/Plasma Kit, no other kit was common between the current study and these studies.

We believe that such a study, among others, is essential in initiating miRNA-based diagnostics and prognostication. The novelty of this study lies in the modification to optimize the manufacturer’s protocol. Our optimized protocol enhanced the miRNA yield of the commercially available miRNeasy Serum/Plasma kit. Combined, it could pave the way for validating miRNA as a cancer biomarker and promoting its use in clinics. We acknowledge that the small sample size limits our study. Studies with a larger sample cohort can add value to the validation of our results. Our study was focused on miRNA extraction from plasma samples, and similar studies across other body fluids are necessary for the widespread use of miRNA in research and diagnostics.

In conclusion, we have demonstrated that miRNeasy Serum/Plasma kit from Qiagen can be reliably used to extract cfmiRNA from small volumes of plasma. We have provided an optimized protocol to improve miRNA isolation further for downstream analyses of mature miRNA.
